# From Clinics to Communities: Understanding Public Perceptions of Dental Services in Pakistan

**DOI:** 10.1111/hex.70177

**Published:** 2025-02-18

**Authors:** Kamran Ali, Daniel Zahra, Ulfat Bashir, Hina Zafar Raja, Asmaa Alkhtib, Minahil Arujj Younas, Ummara Manzoor, Asma Shakoor, Mariya Khalid, Amna Mansoor, Saman Hakeem, Maryam Mumtaz, Mahwish Raja

**Affiliations:** ^1^ QU Health College of Dental Medicine, Qatar University Doha Qatar; ^2^ School of Psychology, Plymouth University Plymouth UK; ^3^ Islamic International Dental College, Riphah International University Islamabad Pakistan; ^4^ CMH Institute of Dentistry Lahore Pakistan; ^5^ Sardar Begum Dental College Gandhara University Peshawar Pakistan; ^6^ KMU Institute of Dentistry Kohat Pakistan; ^7^ Bahria University Health Sciences Karachi Pakistan

**Keywords:** dental services, health services, public perceptions

## Abstract

**Introduction:**

Access to dental services is a core component of public healthcare. The aim of this study was to evaluate the perceptions and experiences of the public regarding access, quality and affordability of dental services in Pakistan.

**Methods:**

It was an analytical cross‐sectional study based on an online survey. The data collection instrument was based on eight items related to participant perceptions and experiences of dental services in public and private sectors. Pretesting of the survey questionnaire was done, and the target participants were approached through social media, and dental service providers.

**Results:**

A total of 1007 participants representing all provinces of the country responded to the study questionnaire including 54.32% (*n* = 547) females and 45.68% (*n* = 460) males. Education and financial status showed the largest effect on perceptions. Although all groups agreed that dental professionals contribute positively to improving public health, those in the ‘No Education’ or ‘Poor’ groups showed fewer positive perceptions about the accessibility, quality, and affordability of dental services. Perceptions of dental services between genders showed minimal differences. Chi‐squared tests of association showed significant (*p* < 0.001) relationships between personal awareness of oral health and respondent characteristics such as education, employment, financial status and location.

**Conclusions:**

This study provides useful insights into the public perceptions and experiences of dental health services in Pakistan. The findings reveal disparities in access, quality, and affordability of dental services among disadvantaged groups, particularly within the public sector. Oral health awareness was also reported to be low amongst people with low educational and financial status. Given the limitations of the current study, further research using qualitative methods may provide a more in‐depth understanding of the facilitators and barriers to dental services to inform a major reform to improve public dental services in the country.

**Patient and Public Involvement and Engagement (PPIE):**

Members of the public with previous experience of using dental services were involved in pretesting of the study questionnaire Pretesting of the survey questionnaire was done in two phases: In the first phase, cognitive interviews were conducted with eight members of the public including four participants each with proficiency in English and Urdu. The purpose of the cognitive interviews was to determine that the participants were able to comprehend all items of the questionnaire accurately. In the second phase the questionnaire was piloted with 15 members of the public who were given a choice to answer the questionnaire in English or Urdu based on their individual preference.

## 1 Introduction

Access to quality and affordable dental services is a critical aspect of overall health care and impacts significantly on individuals' well‐being and quality of life [1, 2]. Dental health is not only an important component of systemic health, but also plays a crucial role in mental and social health, impacting self‐esteem and social interactions [3]. However, access to essential dental services varies widely between developed and underdeveloped countries, leading to significant disparities in oral health outcomes [4]. The burden of oral diseases such as dental caries, and periodontal disease is particularly high for the disadvantaged and poor population groups not only developing but also in developed countries [2]. Access to quality dental services may be impacted by factors related to the population as well as the organisation of the healthcare system [5]. The geographical location, socioeconomic status, and availability of dental professionals may play a crucial part in how people access dental services [6, 7].

Addressing disparities in access and affordability of oral and dental health services is crucial for achieving better oral health outcomes and reducing the global burden of dental diseases. Affordability of dental services is a major concern that affects individuals' ability to seek and receive care. In many developed countries, dental care can be expensive, but well‐structured public health services, insurance schemes, and government subsidies may help mitigate costs for individuals. Disparities in access to dental care may exist amongst disadvantaged communities even in developed countries with strong economies [8].

Dental services are delivered in both the public and private sector in most countries and the governments usually subsidise public sector dental services to make them more affordable for the low and middle‐income populations [9, 10, 11, 12, 13, 14, 15, 16, 17, 18, 19, 20]. A classic example is the National Health Service (NHS) in the UK which has been providing comprehensive dental care services to the public for several decades [21]. However, access to NHS dentists in the UK has been deteriorating in the last few years and seems to have reached a breaking point due to limited funding by the government and unattractive contractual terms for the dentists. The dental contract has been a longstanding issue for the dental profession and the British Dental Association (BDA) and dentists across the country have repeatedly expressed that the existing contract is not fit for purpose and puts the future of NHS dentistry at risk [22, 23]. However, little progress has been made to address the apprehensions of the stakeholders with the result that NHS dentistry is fast reaching a point of crisis. Access to a dentist is becoming increasingly difficult for the public and people have to endure long periods of pain and poor oral health without being able to see a dentist [24, 25].

Pakistan is the fifth most populous country in the world with a population of 24.5 million nearly 40% of the population lives below the poverty line [26]. Political uncertainties, high inflation and lack of long‐term economic policies, economic activity is expected to remain slow, with low GDP growth. Public dental services are available but are not adequate to cater the oral health needs of the population. Despite a remarkable increase in the number of new dental school in recent years and a commensurate increase in the number of new dental graduates, access and affordability of dental care remains challenging for people with low income. The aim of this study was to evaluate the perceptions and experiences of the public regarding access, quality and affordability of dental services in Pakistan.

## 2 Methods

### 2.1 Research Ethics

This research as conducted in accordance with Declaration of Helsinki ethical principles for medical research involving human subjects, including research on identifiable human material and data. Ethical approval was obtained from the ethics review committee of CMH Institute of Dentistry Lahore Pakistan (Approval Number: 615/ERC/CMH/LMC, dated 17/07/2023). Participation was voluntary and all data were recorded and processed anonymously. All participants provided their consent online before starting the survey.

### 2.2 Study Design

This research an analytical cross‐sectional study based on an online survey using Google Forms.

### 2.3 Data Collection Instrument

**Figure 1:**
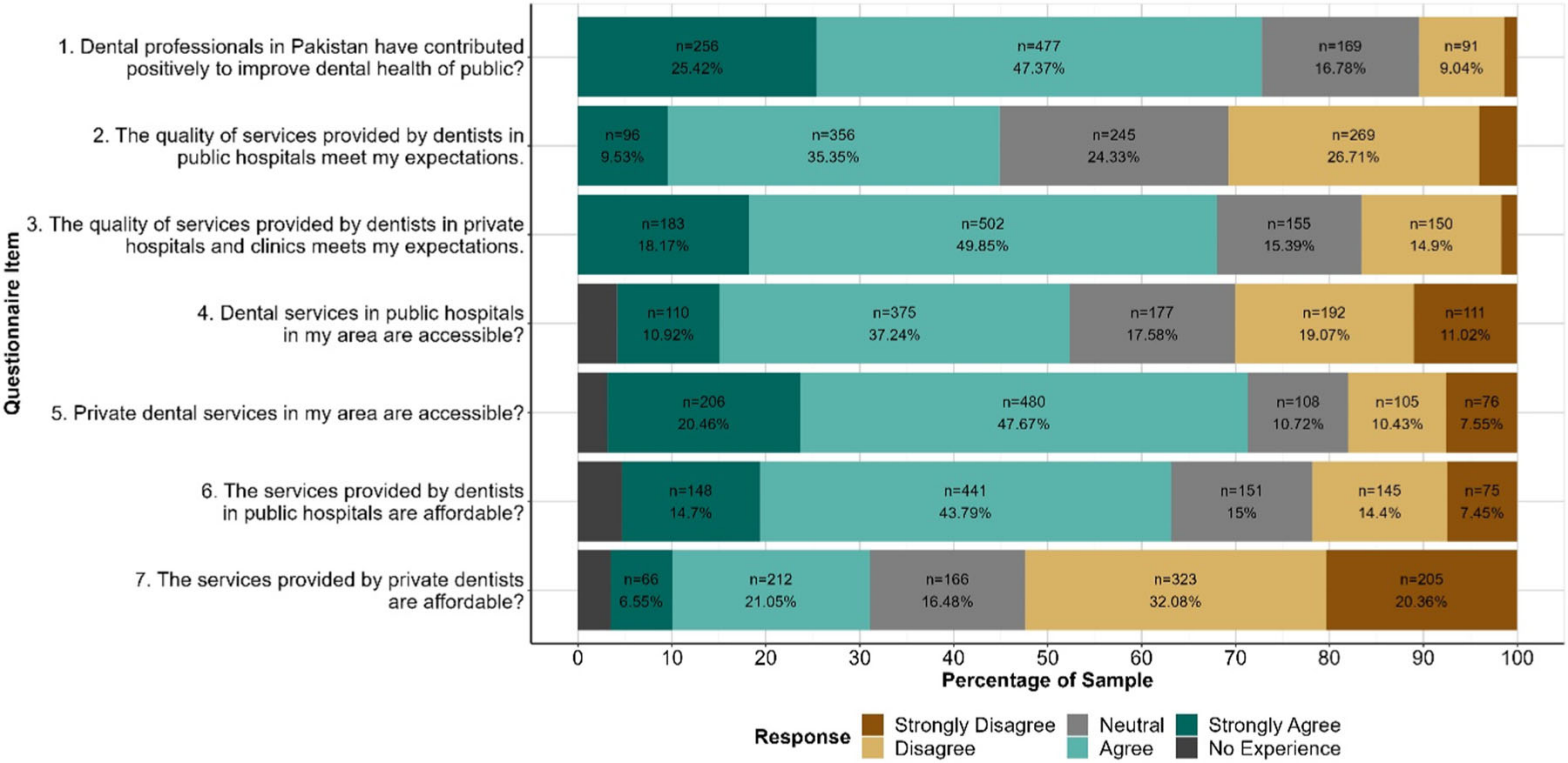
Public perceptions of dental services. Labels omitted from categories accounting for < 5% of responses.

Data collection was based on a survey questionnaire which was developed by a group of dental academics, clinicians, and researchers. The questionnaire consisted of four sections. The first section provided details about the purpose and scope of the project. The second section related to informed consent from participants confirming that their participation was voluntary and that they understood the purpose and scope of the study and that all data related to the study will be processed anonymously. The third section related to demographic information of the participants including age, gender, highest education, location, employment status, and financial status. The fourth section included eight items related to participant perceptions and experiences of dental services in public and private sectors including quality, accessibility, and affordability as depicted in Figure 1. The participants were also asked to rate their own awareness of their oral and dental health.

The questionnaire was initially developed in English language and was translated into Urdu, the national language of Pakistan, by two bilingual members of the research team with Urdu as their first language. The translation was aimed at achieving linguistic and conceptual equivalence, not just literal word‐for‐word translation. Reverse translation of the survey questionnaire in Urdu was done by two members of the research team who were blinded to the original questionnaire in English. Subsequently, the two versions were compared which identified two minor discrepancies which were resolved through discussion by the research team.

Pretesting of the survey questionnaire was done in two phases: In the first phase, cognitive interviews were conducted with eight members of the public including four participants each with proficiency in English and Urdu. The purpose of the cognitive interviews was to determine that the participants were able to comprehend all items of the questionnaire accurately. In the second phase the questionnaire was piloted with 15 members of the public who were given a choice to answer the questionnaire in English or Urdu based on their individual preference. Minimal modifications in the questionnaire were required following pretesting and the questionnaire was finalised with consensus amongst the research team.

### 2.4 Sample Size Calculation

The sample size for this study was determined using a power analysis with G*Power software (version 3.1) [27]. In the Chi‐squared analyses reported, where the degrees of freedom are between 8 and 12, using *α* = 0.05, the minimum sample size required to maintain a power of 0.95, and detect small‐to‐medium effects (*w* = 0.2) was calculated to be *n* = 495. These are also sufficient to detect small effect sizes using analyses such as *t*‐test to compare differences in mean scores between independent groups.

### 2.5 Sampling Technique and Participants

A simple probability sampling technique was used to target adults (18 years or older) belonging to either sex in six regions of the country including Punjab, Sindh, KPK, Balochistan, AJK, and GB. All provinces have a mix of urban and rural populations and Punjab has the highest literacy rate followed by Sindh, KP, Balochistan, AJK and GB. All provinces except GB institutions, which offer undergraduate dental education.

### 2.6 Data Collection

The final version of the questionnaire was administered online using Google Forms. The target participants were approached through popular social media platforms such as Facebook, and Instagram. Additionally, dental service providers in the public and private sectors to participate in an online survey. Data were collected from 15 January 2024 to 14 February 2024.

### 2.7 Data Analysis

All data were analysed and visualised using RStudio incorporating R version 4.0.5. To explore the variation by respondent characteristics and across items, the agreement scale responses were recoded as Strongly Disagree = −2, Disagree = −1, Neutral = 0, Agree = 1, and Strongly Agree = 2. Responses indicating no experience were excluded. Mean scores by subgroups were compared within and across items using mixed analyses of variance, with *t*‐tests or Tukey's HSD for planned pairwise comparisons; means for each characteristic (Gender, Education, Employment, Province, and Financial Status) by Item were also computed.

## 3 Results

### 3.1 Respondent Profile

A total of 1008 participants provided their responses to the online survey. One response was incomplete and was excluded. Of the 1007 responses, 54.32% (*n* = 547) identified as female and 45.68% (*n* = 460) identified as male. Province‐based distribution of demographic factors of participants is summarised in Table 1.

**Table 1:** Demographic characteristics of participants by province.

	Punjab		Sindh		KPK		Balochistan		AJK		GB	
Characteristic	*N*	%	*N*	%	*N*	%	*N*	%	*N*	%	*N*	%
Gender												
Male	315	68.48	57	12.39	41	8.91	33	7.17	9	1.96	5	1.09
Female	387	70.75	61	11.15	62	11.33	23	4.20	13	2.38	1	0.18
Education												
University	361	64.35	91	16.22	73	13.01	20	3.57	13	2.32	3	0.53
College	130	68.06	18	9.42	19	9.95	15	7.85	8	4.19	1	0.52
High School	107	82.31	7	5.38	8	6.15	6	4.62	0	0.00	2	1.54
No Education	67	83.75	2	2.50	1	1.25	10	12.50	0	0.00	0	0.00
Primary School	37	82.22	0	0.00	2	4.44	5	11.11	1	2.22	0	0.00
Employment												
Student	317	76.39	43	10.36	29	6.99	9	2.17	13	3.13	4	0.96
Employed	241	66.76	52	14.40	42	11.63	17	4.71	7	1.94	2	0.55
Full‐Time House Carer	79	63.71	13	10.48	22	17.74	9	7.26	1	0.81	0	0.00
Business	31	55.36	5	8.93	5	8.93	14	25.00	1	1.79	0	0.00
Unemployed	34	66.67	5	9.80	5	9.80	7	13.73	0	0.00	0	0.00
Financial status												
Upper Middle	313	65.21	65	13.54	66	13.75	18	3.75	17	3.54	1	0.21
Lower Middle	231	70.21	42	12.77	30	9.12	19	5.78	4	1.22	3	0.91
Poor	111	83.46	4	3.01	5	3.76	11	8.27	0	0.00	2	1.50
Affluent	47	72.31	7	10.77	2	3.08	8	12.31	1	1.54	0	0.00

Abbreviations: *N*, number; %, percentage.

### 3.2 Overall Perceptions of Dental Services

Overall perceptions of dental services within the sample are shown in Figure 1. Over 72% of participants were positive about the contribution of dentists to promote public dental health. However, less than 45% were satisfied with the quality of dental services in public dental facilities and only 48.16% considered public dental services to be accessible. In contrast, over 68% of participants were satisfied with the quality and accessibility of private dental services. In regard to affordability of dental services, less than 28% of participants considered private dental services to be affordable while public dental services were deemed to be affordable by 58% participants. These findings underscore the need for major improvements in public dental services to meet public expectations

These ratings varied for almost every item by almost every category of respondent information. The *p*‐values from Chi‐squared tests of association between respondent characteristics and item responses are shown in Table 2.

**Table 2:** *p*‐values from Chi‐squared tests of association between respondent characteristics and item responses.

Characteristic	Item 1	Item 2	Item 3	Item 4	Item 5	Item 6	Item 7
Gender	< 0.001	0.009	< 0.001	< 0.001	< 0.001	< 0.001	0.243
Education	< 0.001	< 0.001	< 0.001	< 0.001	< 0.001	< 0.001	< 0.001
Employment	< 0.001	< 0.001	< 0.001	< 0.001	< 0.001	< 0.001	< 0.001
Province	0.027	< 0.001	0.030	0.141	0.380	< 0.001	0.009
Financial Status	< 0.001	< 0.001	< 0.001	< 0.001	< 0.001	< 0.001	< 0.001

*Note:* Item 1: Contribution of dentists to public oral health; Item 2: Quality of dental services in public hospitals meet public expectations; Item 3: Quality of dental services in private hospitals meet public expectations Item 4: Dental services in public hospitals are accessible Item 5: Dental services in private hospitals are accessible; Item 6: Services by dentists in public hospitals are affordable; Item 7: Services by private dentists are affordable.

### 3.3 Perceptions by Respondent Characteristics

**Figure 2:**
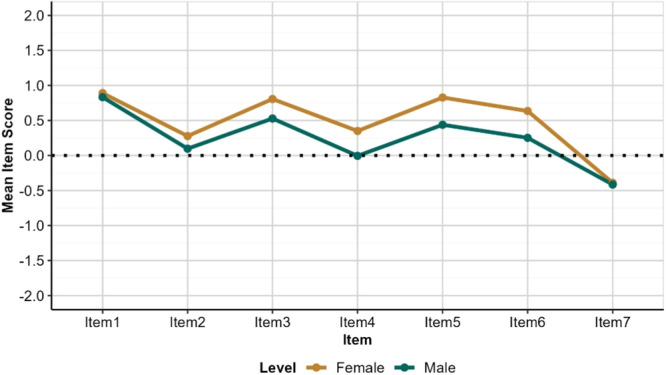
Mean item scores by gender. Higher scores were observed for females indicating more positive perceptions about the quality and ease of access to dental services in public and private sectors. *Item 1: Contribution of dentists to public oral health; Item 2: Quality of dental services in public hospitals meet public expectations; Item 3: Quality of dental services in private hospitals meet public expectations Item 4: Dental services in public hospitals are accessible Item 5: Dental services in private hospitals are accessible; Item 6: Services by dentists in public hospitals are affordable; Item 7: Services by private dentists are affordable.

There seems to be minor difference in perceptions of dental services between genders. Although the mean score for males is lower than females on Items 2, 3, 4, 5, and 6, the pattern of responses across items for both genders are broadly comparable as shown in Figure 2.

**Figure 3:**
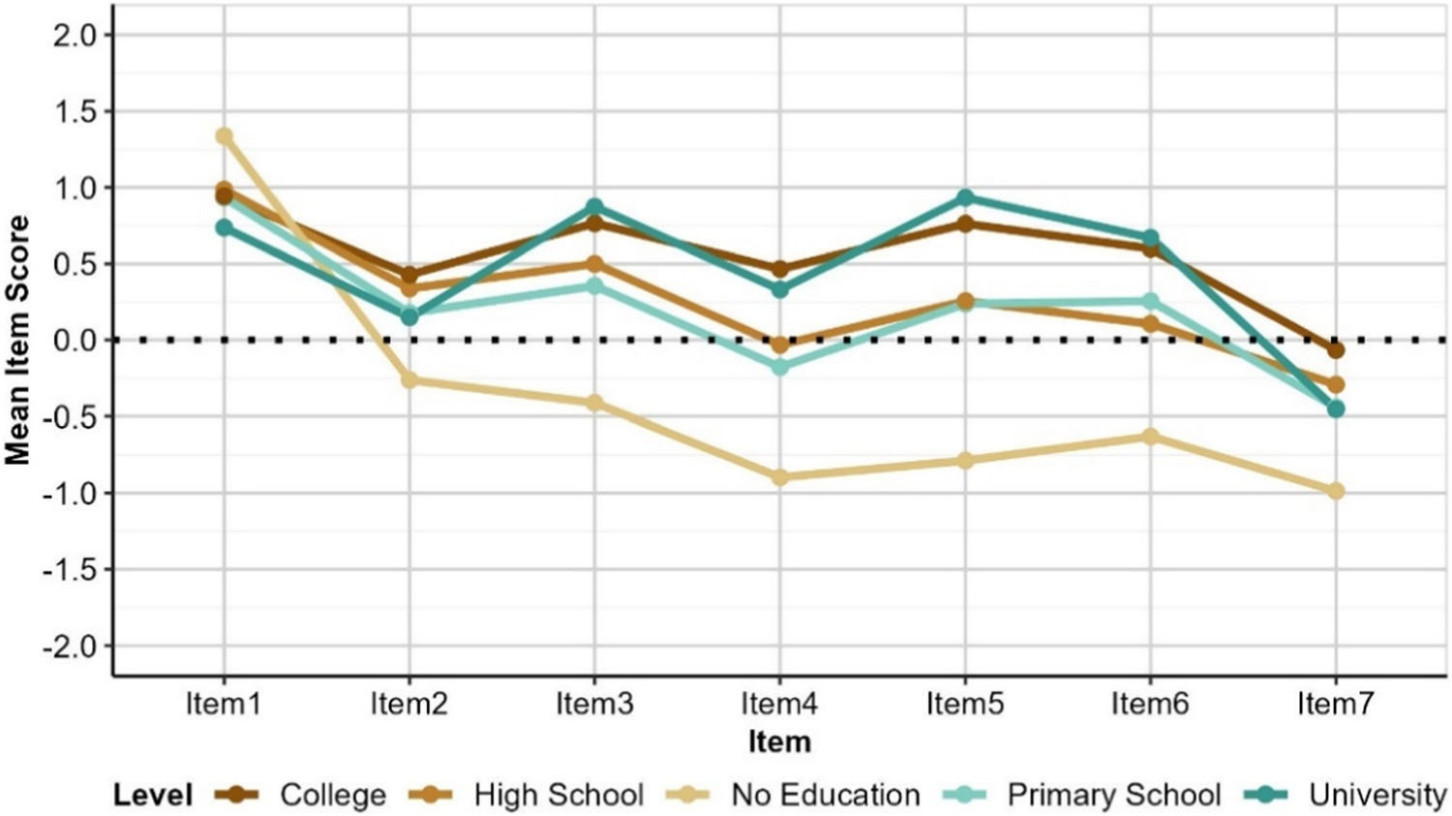
Mean item scores by education. Lowest mean scores were observed for participants with “No education” indicating fewer positive experiences of dental services in public and private sector. *Item 1: Contribution of dentists to public oral health; Item 2: Quality of dental services in public hospitals meet public expectations; Item 3: Quality of dental services in private hospitals meet public expectations Item 4: Dental services in public hospitals are accessible Item 5: Dental services in private hospitals are accessible; Item 6: Services by dentists in public hospitals are affordable; Item 7: Services by private dentists are affordable.

**Figure 4:**
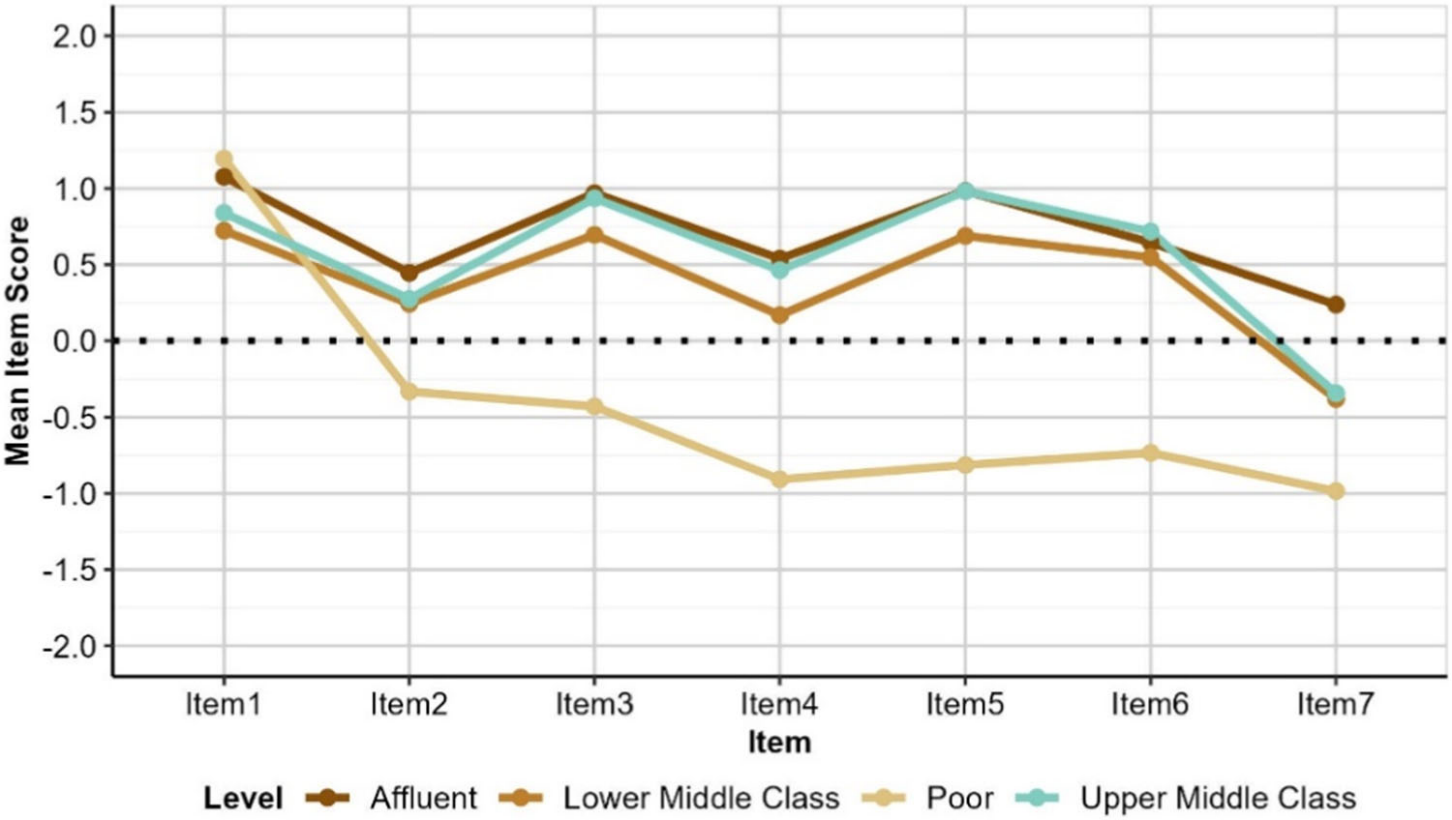
Mean item scores by financial status. Lowest mean scores were observed for participants who identified themselves as “Poor” indicating fewer positive experiences of dental services in public and private sector. *Item 1: Contribution of dentists to public oral health; Item 2: Quality of dental services in public hospitals meet public expectations; Item 3: Quality of dental services in private hospitals meet public expectations Item 4: Dental services in public hospitals are accessible Item 5: Dental services in private hospitals are accessible; Item 6: Services by dentists in public hospitals are affordable; Item 7: Services by private dentists are affordable.

Education and Financial Status have the largest effect on perceptions as depicted in Figures 3 and 4, respectively. Although all groups agree that dental professionals contribute positively to improving public health (Item 1), those in the ‘No Education’ or ‘Poor’ groups show notably lower levels of agreement for Items 2, 3, 4, 5, 6, and 7. This suggests that these groups rated the services provide by public and private hospitals as lower quality (Items 2 and 3), less accessible (Items 4 and 5), and less affordable (Items 6 and 7) than other groups. This is not likely to be skewed by a lack of experience with private services; the numbers of those responding ‘no experience’ across Educational and Financial Status groups is minimal, and comparable.

**Figure 5:**
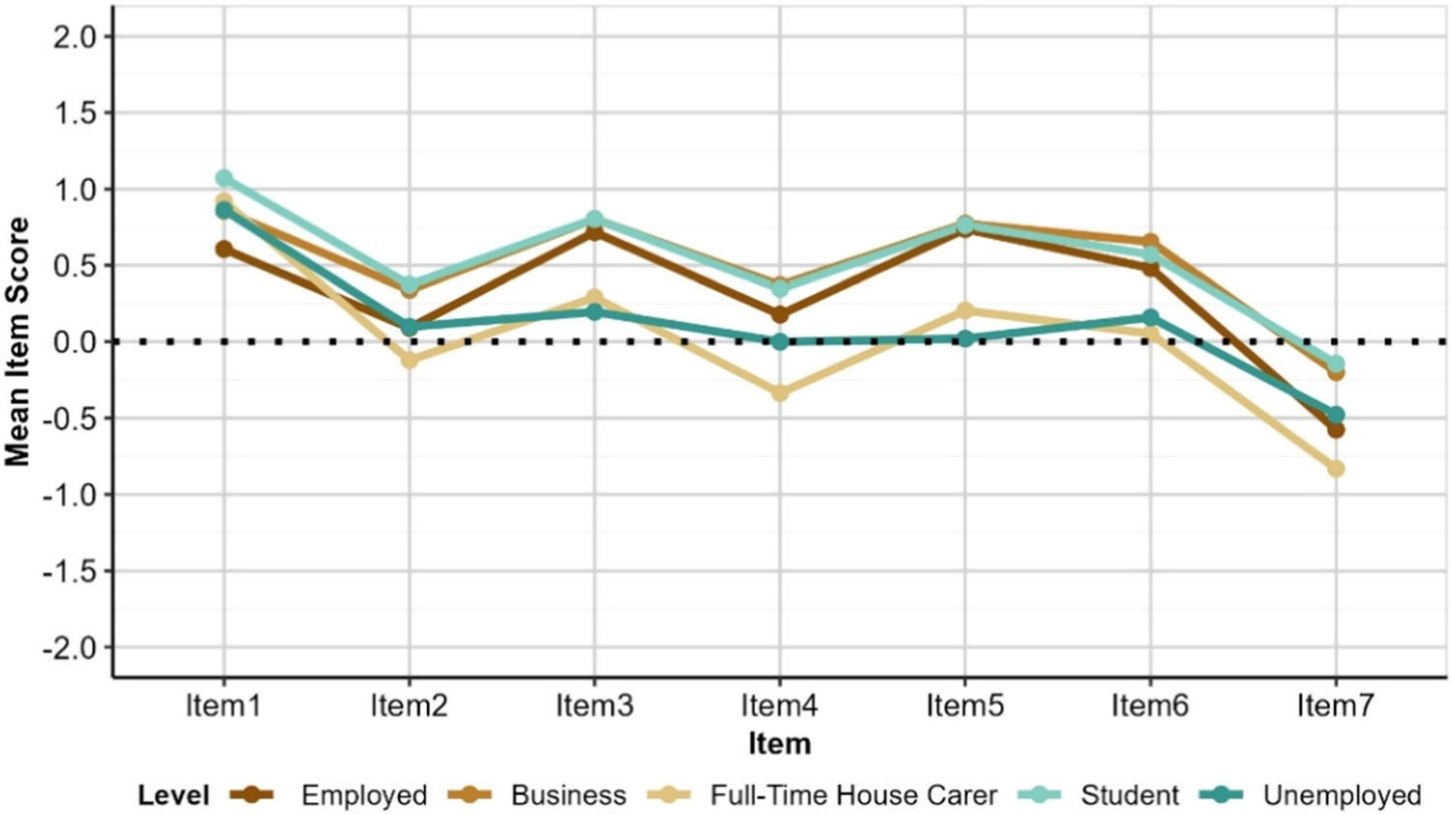
Mean item scores by employment status. Lowest mean scores were observed for participants who identified themselves as “Full time house carers” and “Unemployed” indicating fewer positive experiences of dental services in public and private sector. *Item 1: Contribution of dentists to public oral health; Item 2: Quality of dental services in public hospitals meet public expectations; Item 3: Quality of dental services in private hospitals meet public expectations Item 4: Dental services in public hospitals are accessible Item 5: Dental services in private hospitals are accessible; Item 6: Services by dentists in public hospitals are affordable; Item 7: Services by private dentists are affordable.

Employment Status shows a similar but far less pronounced pattern, with ‘Full‐Time House Carers’ and ‘Unemployed’ respondents reporting experiencing public and private care as lower quality, less accessible, and less affordable than other groups (Figure 5). These ratings, whilst lower, are much closer to those of other Employment Status groups than is the case for ‘No Education’ and ‘Poor’ relative to other Education and Financial Status groups.

**Figure 6:**
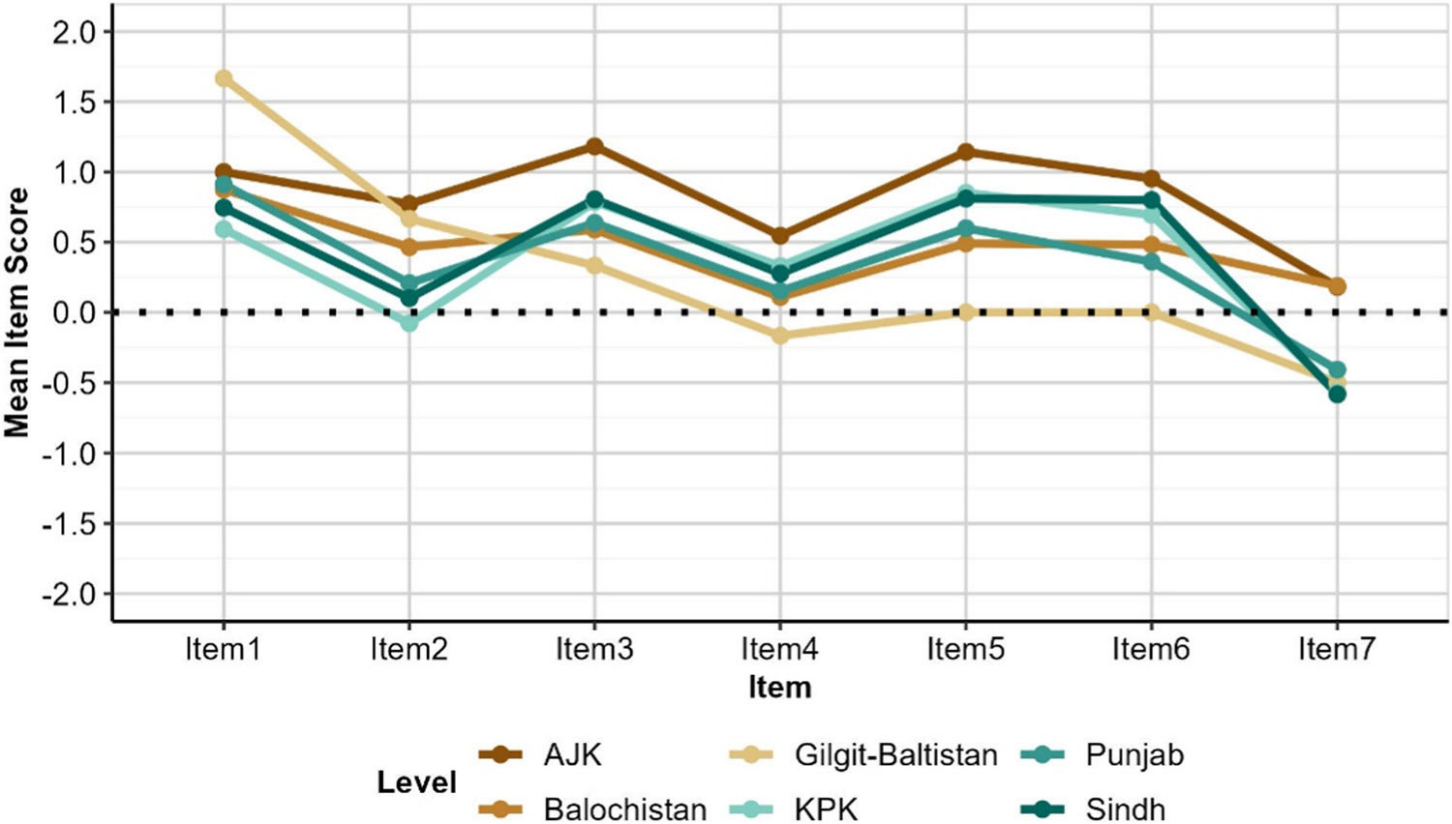
Mean item scores by province. Highest mean scores were reported by participants from AJK while lowest mean scores were reported by participants from Gilgit‐Baltistan. *Item 1: Contribution of dentists to public oral health; Item 2: Quality of dental services in public hospitals meet public expectations; Item 3: Quality of dental services in private hospitals meet public expectations Item 4: Dental services in public hospitals are accessible Item 5: Dental services in private hospitals are accessible; Item 6: Services by dentists in public hospitals are affordable; Item 7: Services by private dentists are affordable.

Province also appears to have limited consistent impact of perceptions as shown in Figure 6. Although responses from GB are lower for multiple items, these averages are across only six respondents.

### 3.4 Self‐Awareness Scale

**Figure 7:**
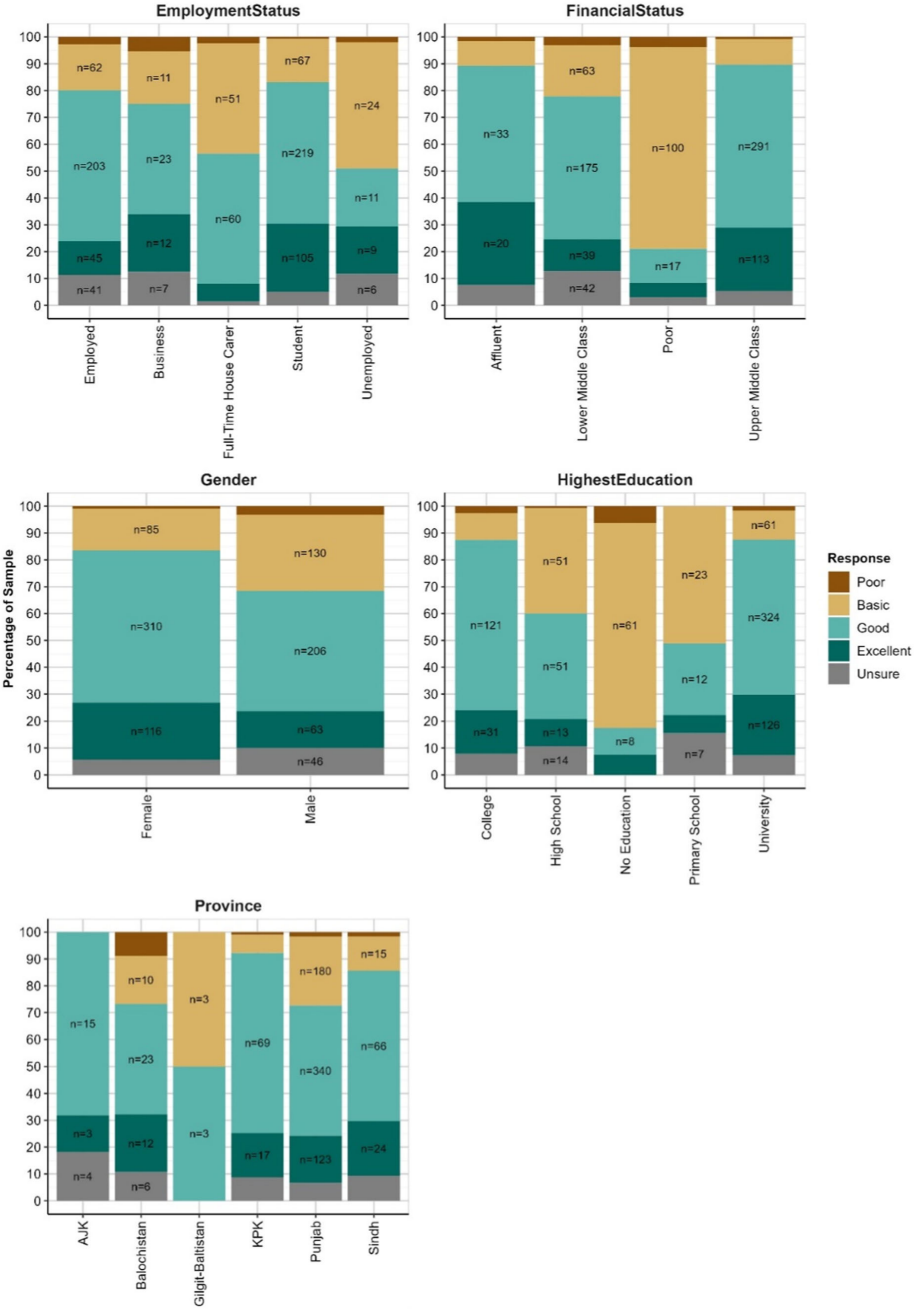
Self‐awareness ratings by characteristics. Labels have been omitted for groups < 10%. Respondents in the ‘Full‐Time House Carer’, ‘Unemployed’, ‘Poor’, ‘No Education’, and ‘GB’ groups reported lower levels of awareness regarding dental and mouth health.

Chi‐squared tests of association show significant (*p* < 0.001) relationships between levels of all respondent characteristics and responses to the item ‘How would you rate your self‐awareness regarding your mouth and dental health’. Distributions of these responses by characteristics are shown in Figure 7. Awareness shows similar patterns to perceptions of dental services. Respondents in the ‘Full‐Time House Carer’, ‘Unemployed’, ‘Poor’, ‘No Education’, and ‘GB’ groups typically indicate having poorer or more basic self‐awareness regarding dental health, and to a lesser extent this is also apparent in ‘High School’ and ‘Primary School’ education groups relative to ‘College’ and ‘University’ education groups, within which a far higher proportion report their awareness as ‘Good’ or ‘Excellent’.

## 4 Discussion

This study to explores the perceptions and experiences of public regarding access affordability and quality of dental services in Pakistan, one of the most populous countries in the world. Although majority of the participants valued the contribution by dentists, less than 50% were satisfied with the quality and accessibility of public dental services compared to 68% satisfaction with private dental services. These findings are consistent with other studies which also show higher satisfaction with private dental services [28]. Higher satisfaction rates with private dental services may be related to a number of factors including easier access, reduced waiting times, and the range of treatments offered to patients [29]. In regard to affordability of dental services, less than 28% participants considered private dental services to be affordable while public dental services were deemed to be affordable by 58% participants. These findings mirror patient perceptions globally as private dental services are widely reported to be more expensive [30, 31, 32]. Overall the frequencies of “no experience” of public and private dental services were relatively low. This may be explained by the fact that public dental services are oversubscribed and it is not unusual for public to utilise private dental services during emergency situations such as acute pain or infection.

Significant differences in the perceptions and experiences of the participants were observed in relation to educational level and financial status. Participants with lower levels of education and financial status reported poor experiences in regard to quality, accessibility and affordability of dental services. Participants from GB also reported fewer positive experiences of dental services. GB is a remote area in the northern region of the country with minimal economic activity apart from tourism. It is worth noting that the same groups of participants also rated their levels of oral health awareness to be low. These findings are in accord with published literature and highlight the need for a renewed focus on providing affordable dental services to disadvantaged groups of population [13, 32]. Currently, people with a low financial status do not have appropriate opportunities for health insurance and making improvements in public sector dental services seems to be the only feasible option for these groups. Females participants rated their awareness of oral hygiene better than males, although the results were statistically not significant. These differences corroborate with previous studies on oral health behaviours in men and women [33, 34, 35].

It is widely reported in the literature that disadvantaged communities across the globe have inadequate access to health services including dental care [1, 4, 5, 6, 12, 36, 37, 38, 39, 40]. However, given the population of the country, the number of people living below the poverty line is on the rise and the scale of people with inadequate healthcare has reached unprecedented levels. It is also pertinent to mention that Pakistan has one of the highest incidence and prevalence of oral cancer in the world and is often diagnosed at an advanced stage due to delayed referral [41, 42, 43]. Dentists are recognised to play a crucial role in the prevention and recognition of oral cancer as dental visits provide opportunistic cancer screening opportunities [44]. Therefore, access to dental care is of fundamental importance not only to prevent common oral diseases like dental caries, and periodontal disease but also for prevention and prompt recognition of oral cancerous lesions.

It is important to delve into the underlying reasons for poor access to quality dental services in Pakistan. The number of dental colleges and graduating dentists in Pakistan has seen a remarkable increase in the last 20 years. Just before the turn of the millennium, there were only six dental colleges across the country, all in the public sector. Subsequently, a rapid growth of dental colleges was observed in both public and private sector and now there are 68 institutions offering an undergraduate dental qualification. However, this increase in new dental graduates is not reflected in improved public access to dental services. There are several reasons for suboptimal public perceptions and experiences regarding access and affordability of dental services. First, a vast majority of dental graduates tend to settle in large urban cities and engage in private dental practice due to a better income potential and quality of life. Second, the economy of Pakistan is unstable with a rising foreign debt which places significant financial constraints on the government to maintain and improve public dental services. Moreover, the rapid growth of population in Pakistan has affected human development and quality of life negatively [45, 46]. The aforementioned factors pose barriers to the availability of affordable dental services to the masses, particularly in small towns and rural communities.

A fundamental challenge in Pakistan is lack of visible planning of healthcare workforce by the government [47]. Gaps in strategic and operational planning have a negative impact on the quality of health services, particularly in the public sector [48]. A rapid increase in private medical and dental institutions seems to be largely driven by financial interests as the high tuition fee translates into significant profit for the investors. However, there is lack of clarity regarding a robust plan to align the number of new medical and dental graduates with the public need and/or employment opportunities [49, 50]. Similar challenges are reported from India where a rapid growth of medical and dental colleges in the private sector has raised concerns regarding quality assurance of medical education and patient safety [51, 52, 53]. It is recognised that more dental graduates and dental care professionals such as dental hygienists and therapists are required to meet the oral healthcare needs of the population. However, relevant government and professional bodies must work together to develop comprehensive strategies regarding effective deployment of new graduates to maximise the benefits to the public. Relevant government bodies and professional organisations need to develop and deliver oral health education to the masses. And may use social media platforms as a cost‐effective and influential means of communication to promote oral health [54, 55, 56].

There are several limitations related to the sampling and recruitment of participants in this study which limit the generalisability of the findings and this study should be best treated only as a snapshot of the public perceptions of dental services in a populous country. Pakistan is a large country with markedly heterogeneous demographics, especially socioeconomic status of the people, and availability of healthcare services. The study used an online questionnaire and it is possible that remote areas with limited internet connectivity may be underrepresented. The study utilised social media for recruitment of participants and the higher percentage of responses from students suggests that the participation is skewed towards younger people. This may be related to more frequent use of social media by young people and therefore, age of participants could have potentially confounded the findings of the study. Other demographic variables such as education, employment, financial status and location of the participants could have similar effects on the mean scores of the participants which may limit the generalisability of the findings. It is also acknowledged that use of Likert scale to categorise and score responses from the participants also has some drawbacks. Although Likert scales are widely used for their simplicity and ease of interpretation, the numerical values assigned to responses (e.g., −2 for “Strongly Disagree” to +2 for “Strongly Agree”) do not inherently represent true intervals of measurement. The perceived distance between points may vary among respondents, making the scale subjective and limiting its precision. This arbitrariness necessitates caution when interpreting these scores. Focussed studies on specific communities using stratified sampling techniques are required to gain a more comprehensive understanding about the access and quality of dental services. Moreover, the survey questionnaire only consisted of closed‐ended items and use of qualitative methods such as focus groups and interviews may also provide a more in‐depth understanding of the facilitators and barriers to dental services.

## 5 Conclusion

This study provides useful insights into the public perceptions and experiences of dental health services in Pakistan. The findings reveal disparities in access, quality, and affordability of dental services among disadvantaged groups, particularly within the public sector. Oral health awareness was also reported to be low amongst people with low educational and financial status. Given the limitations of the current study, further research using qualitative methods may provide a more in‐depth understanding of the facilitators and barriers to dental services to inform a major reform to improve public dental services in the country.

## Author Contributions

**Kamran Ali:** conceptualization, methodology, writing–review and editing, writing–original draft, project administration, supervision, validation. **Daniel Zahra:** formal analysis. **Ulfat Bashir:** data curation, investigation. **Hina Zafar Raja:** investigation. **Asmaa Alkhtib:** conceptualization, writing–review and editing, resources, investigation. **Minahil Arujj Younas:** data curation, resources, investigation. **Ummara Manzoor:** data curation, resources, investigation. **Asma Shakoor:** investigation. **Mariya Khalid:** investigation. **Amna Mansoor:** investigation. **Saman Hakeem:** investigation. **Maryam Mumtaz:** investigation. **Mahwish Raja:** investigation, methodology, writing–review and editing, data curation.

## Ethics Statement

This research as conducted in accordance with the Declaration of Helsinki ethical principles for medical research involving human subjects, including research on identifiable human material and data. Ethical approval was obtained from the ethics review committee, CMH Institute of Dentistry Lahore Pakistan (Approval Number: 615/ERC/CMH/LMC, dated 17/07/2023). Participation was voluntary and all data were recorded and processed anonymously. All research data were stored following university data protection regulations.

## Consent

Informed consent was obtained from all participants in the study.

## Conflicts of Interest

The authors declare no conflicts of interest.

## Data Availability

The data underlying this article will be shared on reasonable request to the corresponding author.
